# Hydration, Refinement, and Dissolution of the Crystalline Phase in Polyamide 6 Polymorphs for Ultimate Thermomechanical Properties

**DOI:** 10.1021/acs.macromol.2c00211

**Published:** 2022-06-13

**Authors:** Milo Gardeniers, Mohanraj Mani, Ele de Boer, Daniel Hermida-Merino, Robert Graf, Sanjay Rastogi, Jules A. W. Harings

**Affiliations:** † Aachen-Maastricht Institute for Biobased Materials, Maastricht University, P.O. Box 616, 6200 MD Maastricht, The Netherlands; ‡ European Synchrotron Radiation Facility (ESRF), DUBBLE-CRG, FR-38043 Grenoble Cedex, France; § Max Planck Institute for Polymer Research, Ackermannweg 10, 55128 Mainz, Germany; ∥ King Abdullah University of Science and Technology, 4700 KAUST, Thuwal 23955-6900, Saudi Arabia; ⊥ Departamento de Física Aplicada, CINBIO, Universidade de Vigo, Campus Lagoas-Marcosende, E36310 Vigo, Galicia, Spain

## Abstract

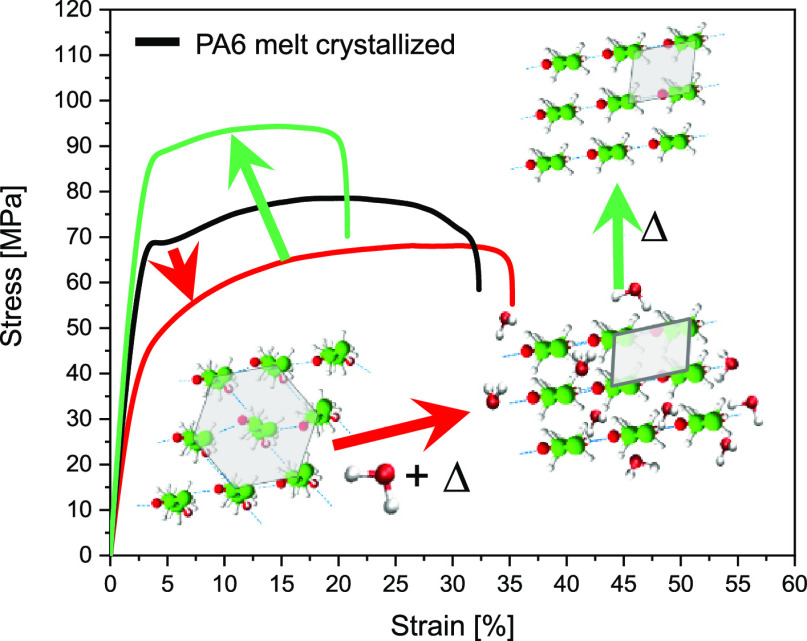

Timescales of polyamide 6 melt-shaping technologies, relative to the dynamics of conformational rearrangements upon crystallization, challenge the formation of the most thermodynamically favorable chain packing and thus optimum performance. In this publication, we make use of the mediation of hydrogen bonding by water molecules in the superheated state of water, i.e., above 100 °C in a closed environment, in the structural refinement of polyamide 6 for enhanced thermomechanical performance. The paper addresses dissolution and (re)crystallization of different polyamide 6 polymorphs in the superheated state of water by time-resolved simultaneous small- and wide-angle X-ray scattering and solid-state ^1^H NMR spectroscopy and the effect on mechanical properties. The experiments reveal that upon heating in the superheated state of water, the pseudo-hexagonal phase dissolves at relatively low temperature and instantly crystallizes in a defected monoclinic phase that successively refines to a perfected monoclinic structure. The dissolution temperature of the pseudo-hexagonal phase of polyamide 6 is found to be dependent on the degree of crystal perfection originating from conformational disorder and misalignment of hydrogen bonding in the lattice, retrospectively, to the Brill transition temperature. The perfected monoclinic phase below the dissolution temperature can be preserved upon cooling but is plasticized by hydration of the amide moieties in the crystalline phase. The removal of water from the hydrated crystals, in the proximity of Brill transition temperature, strengthening the hydrogen bonding, occurs. Retrospectively, the most thermodynamically stable crystallographic phase is preserved and renders an increase in mechanical properties and dimensional stability of the product. The insight obtained on the influence of superheated water on the structural refinement of imperfected crystallographic states assists in polyamide 6 postprocessing strategies for enhanced performance.

## 1 Introduction

Polyamides, being natural or synthetic, have been known for their diverse role in (bio)engineering. Their thermomechanical performance relies on conformational amide stiffness and intra- and intermolecular hydrogen bonding. While hydrogen bonding in the amorphous phase mainly affects postyield deformation, hydrogen bonding in the crystalline domains contributes among other structural and morphological parameters to the initial stiffness and absolute stress level.^1,2^ Stiffness, strength, and melting temperature scale with the strength of the hydrogen bond. Consequently, the amide density, the repetitive spatial position along the polymeric chain that progresses to adjacent chain segments upon crystallization, and their spatial planarity are decisive factors in the thermomechanical performance.^3−5^

**Figure 1:**
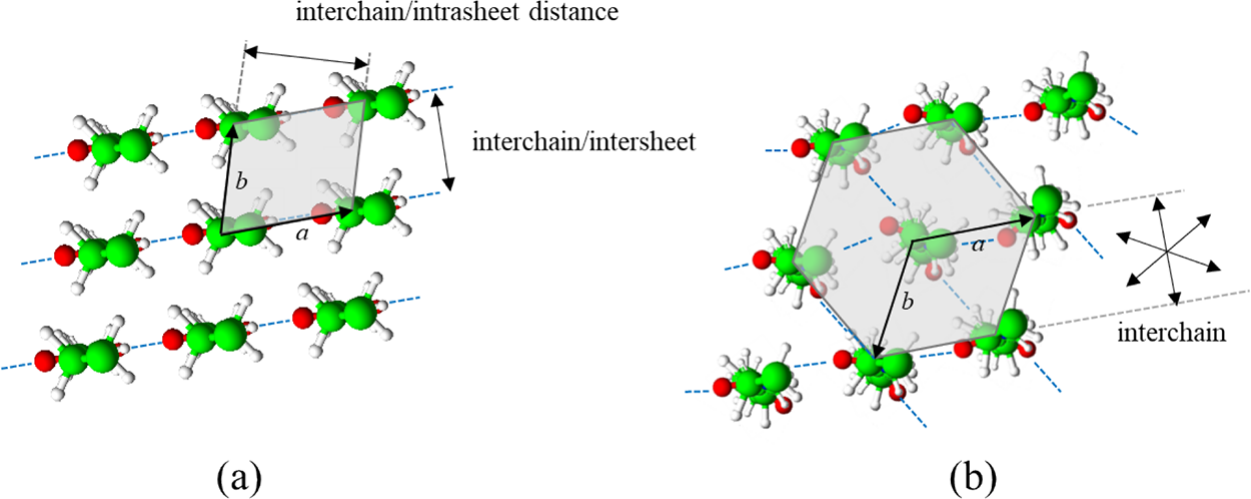
Projection of the molecular organization of polyamide 6 along the *c*-axis for (a) the monoclinic and (b) pseudo-hexagonal crystallographic forms in which the *a*–*b* planes of the unit cells and the defined interchain d-spacings are defined.

In the most thermodynamically stable crystalline structure, the hydrogen bonds are uniplanar with the amide moieties spatially positioned at a proximity where the electron exchange between the amide moieties is optimum, forming hydrogen-bonded sheets that stack into a three-dimensional lattice by Van der Waals forces.^3,6^ This organization defines interchain/intrasheet and interchain/intersheet planes in mostly monoclinic or triclinic unit cells.^3^ Upon increasing temperature, the two characteristic diffraction signals tend to merge, which leads to a pseudo-hexagonal packing prior to melting and is referred to as the Brill transition.^7^ High cooling rates may lead to crystal imperfection and pseudo-hexagonal structures existing at temperatures below the glass transition temperature, as recently demonstrated by fast scanning chip calorimetry experiments and successive FTIR and X-ray diffraction.^8^ The origin of the Brill transition in even-even polymers has been enlightened by temperature-dependent wide-angle X-ray diffraction and complemented by detailed FTIR and solid-state NMR spectroscopic methods.^9−13^ The molecular organization of polyamide 6, the hydrogen bonding directionality of polyamide 6 in the monoclinic and pseudo-hexagonal crystallographic packing, and the defined interchain d*-*spacings are illustrated by projection along the *c*-axis of the unit cells in Figure 1.

The Brill transition relies on the thermally induced transition of methylene trans conformers into gauche conformers similar to that observed in atactic polyacrylonitrile.^13,14^ Upon heating, the heterogeneity of methylene conformers gains in amplitude, transferring the rotational motion to the yet effective intrasheet hydrogen bonding moieties as described by Tashiro and Yoshioka^15^ and English et al.^11,16^ and causing the interactive hydrogen bonding moieties to wag out of plane. In combination with the liberational motion of the methylene conformers that tend to escape from the crystal along the chain axis, the intrasheet distance decreases. Simultaneously, thermal expansion causes an increase in intersheet distance. A random distribution of the hydrogen bonding planes results in a high-symmetry pseudo-hexagonal phase. Recently, Lotz recognized the distinction of pleated and rippled sheet structures of even-even polyamides with short methylene sequences,^17^ enabling a systematic conformational and structural analysis on the origin of the Brill transition.^18^ It was concluded that the Brill transition originates in a temperature-induced dynamic interconversion between mirror image conformers, validating the discussion to define the Brill transition on the molecular scale instead of the crystallographic length scale.

The Brill transition of even-even polyamides, particularly polyamide 46, in the presence of water was reported. By calorimetric and synchrotron X-ray scattering methods, dissolution without hydrolysis was observed close to the Brill transition temperature.^19−23^ In their superheated state, water molecules experience low intermolecular forces and high mobility.^24^ To disclose the origin of polyamide dissolution in the superheated state of water, ^1^H HR-MAS NMR provided insight into the interaction of water molecules with the hydrogen bonding motifs of polyamide 46.^13^ On heating in the presence of water, two different states of mobility for water molecules are observed prior to the sharpening of, first, the NH protons and, second, the aliphatic protons. The sharpening of the polyamide proton bands suggests sudden enhancement in the mobility of the polymer chains, which has been explained by full dissolution of the semicrystalline polymer. While on cooling, with crystallization, the chain mobility decreases and two different mobile phases of water prevail. These findings are supported by WAXD studies where, on heating, the crystalline diffraction peaks disappear upon dissolution^20^ and reappear with crystallization in water.^21^ Lath-shaped single crystals, equivalent to those grown from 1,4-butanediol, were obtained from water solution^21^ (Figure 3). These studies were extended to other polyamides. Up to about 75 wt % polyamide, the dissolution temperature is independent of its weigh fraction, following Flory–Huggins theory on dissolution of polymers in a good solvent.^21^

Interaction of superheated water with the amide moieties has been reported to mediate crystal refinement under quiescent,^21,25,26^ processing,^27,28^ and postprocessing conditions.^2,22,23^ Like strength, stiffness, and melting temperature, the depression of the melting temperature scales with the amide density, or chain polarity, as reported for polyamide 6, 66, 612, and 12 by Evans and Lesser.^5^ Inclusion of the melting temperature depression of polyamide 46 and the biobased polyamides 69 and 109 as measured by Vinken et al.^20,21^ and Tao et al.,^29,30^ respectively, reveals a linear relationship (Figure S1). However, the melting temperature depression of polyamide 6 and 66, albeit characterized by the same amide density, differs remarkably. This difference suggests that chain packing in different crystallographic phases next to polarity affects the dissolution temperature. In all reported studies, the influence of a controlled crystallographic phase on annealing and dissolution in the superheated state of water, specifically above 140 °C, remained elusive. It is known that the Brill transition temperature, and hypothetically so, the aqueous solubilization temperature, depends on the crystallization conditions and resulting chain packing. In fact, Pepin et al. recently reported crystallographic and calorimetric evidence for a Brill transition in the pseudo-hexagonal β form of polyamide 6 just above 100 °C.^31^

Relative timescales of the conformational rearrangement determine the crystallographic phase and degree of crystal perfection or deviations thereof. For polyamide 6, multiple possibilities in spatial organization induce polymorphism.^32−34^ On melt processing, typically two distinct crystallographic classifications coexist at room temperature. The first and most thermodynamically stable phase is the monoclinic (α) phase, which is typically obtained on slow cooling from melt, <5 °C/s, or isothermal crystallization above 157 °C that are industrially not appreciated.^33^ Crystallization in the presence of superheated water at cooling rates of 10 °C/min and higher leads to instant formation of the monoclinic phase.^20,21^ In the monoclinic phase, linearly aligned hydrogen bonds are formed between antiparallel chains that are in an all-trans chain conformation. The stacking of hydrogen-bonded sheets gives a three-dimensional lattice that leads to strong interchain/intrasheet (200) and interchain/intersheet (002) diffraction signals. The crystal perfection index (CPI) is a measure to quantify the thermodynamic stability of the monoclinic phase based on the relative positions of the (200) and (020) diffraction signals,^35^ distinguishing defected lattices (CPI < 1) from a perfect lattice (CPI = 1) denoted as α_2_ and α_1_, respectively. The thermodynamically less stable symmetric pseudo-hexagonal phase, β, is able to accommodate gauche conformers with random hydrogen bonding planes, which are typically formed at high crystallization or cooling rates.^33,34^ Due to the non-unidirectionality of the hydrogen bonding planes, only one interchain spacing (200,002) is observed. At low temperature, the conformational motion of the methylene segments in the crystals is limited, but upon heating, an increase in kinetic energy (kT) induces more conformational motion of the methylene units. The resulting high-temperature crystallographic phase is an analogue to the pseudo-hexagonal phase observed above the Brill transition. The other reported polymorphs of polyamide 6 are derivations of the monoclinic and pseudo-hexagonal structures.

With respect to the monoclinic structure, the Brill transition temperature and *E* modulus of the pseudo-hexagonal phase are considerably lower. Based on an X-ray method and linear compressibility in three dimensions, Tashiro and Tadokoro calculated the theoretical modulus of the monoclinic lattice to be six times larger than that of the pseudo-hexagonal lattice.^36^ Experimentally, using nanoindentation, a factor of 2 difference in the modulus and hardness was reported.^37^ From tensile studies on dried and humidified polyamide 6 samples, Miri et al. concluded that crystal plasticity in humidified conditions is augmented by the water-induced unzipping of hydrogen bonds at a defective crystal interface, which was found to be more abundantly present in the low-temperature pseudo-hexagonal phase.^32^ To exploit the water-mediated crystal refinement of polyamide 6 during processing, the crystal phase stability and timing of crystallographic changes in superheated water are pivotal. In this study, we demonstrate a route to achieve the thermodynamically stable monoclinic phase via reversible hydration of the crystals. We will investigate the stability, dissolution, and crystallization of different polyamide 6 polymorphs in the presence of water in the lower-temperature regime of the superheated state below 145 °C and even below 100 °C. We follow the crystallographic and structural changes by time-resolved simultaneous synchrotron wide-angle X-ray diffraction and small-angle X-ray scattering. Localization of water molecules in different chemical environments is followed by *in situ* solid-state ^1^H NMR spectroscopy. The molecular insight enhances our fundamental understanding of water-induced structure evolution influencing the mechanical properties in polyamide 6 as well as in hydrogen-bonded polymers in general.

## 2 Experimental Section

### 2.1 Polyamides and Sample Preparation

Melt-processed polyamide 6 granulate (Akulon), having a weight-average molecular weight of 74,000 g/mol as measured by gel permeation chromatography (GPC) against PMMA standards, was kindly supplied by DSM (Geleen, The Netherlands). To determine potential changes in molecular weight upon the various (hydro)thermal treatments, GPC was carried out on a PSS SECcurity GPC system using the Agilent 1260 Infinity instrument technology. The apparatus was equipped with a PFG Combination precolumn and two PFG Combination microcolumns. Distilled HFIP containing 0.019% sodium trifluoroacetate was used as eluent using a 0.3 mL/min flow rate at 40 °C. The sample weight was kept constant at 5 ± 0.2 mg.

For ease in diffusion of water molecules, the granulate of the as-received polyamides was cryogenically milled using a Fritsch Pulverisette 14 equipped with a 0.5 mm sieve. The sieved fraction with a particle size ranging from 0.4 to 0.5 mm was used for *in situ* X-ray scattering and solid-state NMR spectroscopy experiments. The Brill transition, melting, and aqueous solubilization were studied for perfected monoclinic (α_1_) and pseudo-hexagonal (β) phases as defined by Pepin et al.^31^ The monoclinic α_1_ phase was made by crystallization from the superheated state of water using 30 wt % polyamide 6 and a cooling rate of 10 °C/min. The pseudo-hexagonal phase was obtained by quenching polyamide 6 from melt (250 °C) at −78 °C using a dry-ice acetone mixture and successive annealing at 80 °C. Defected monoclinic samples were prepared by annealing the quenched samples at 190 °C for 1 h.^31^ The coexisting defected monoclinic phase (α_2_) and pseudo-hexagonal β phase, also referred to as the melt-processed polyamide 6, were generated by cooling the polyamide 6 melt (240 °C) to room temperature at 200 °C/min. Procedures of sample preparation, crystallographic description, and sample notation are summarized in Table 1.

**Table 1:** Sample Notation Based on the Method of Preparation and Crystallographic Description

preparation	crystallographic phase	crystallographic notation	sample notation
superheated water-crystallized	perfected monoclinic (CPI = 1)	α_1_	wc
melt-processed/crystallized	defected monoclinic (CPI < 1) and pseudo-hexagonal	α_2_ and β	mc
quenched from melt	amorphous		
quenched from melt and annealed at 80 °C	pseudo-hexagonal	β	
quenched from melt and annealed at 190 °C	defected monoclinic	α_2_	

### 2.2 Synchrotron Small-Angle X-ray Scattering and Wide-Angle X-ray Diffraction (SAXS/WAXD)

The crystallographic and morphological changes during the Brill transition, melting, and aqueous solubilization were investigated at the DUBBLE beamline (BM26B) of the European Synchrotron Radiation Facility (ESRF) in Grenoble, France. The 200 × 200 μm^2^ X-ray beam with a wavelength of 1.033 Å (12 keV) was employed to follow the diffraction patterns at small and wide angles.^38,39^ All powdered samples, whether or not immersed in water, were placed in glass capillaries, closed in an in-house-designed pressure cell and brought reversibly into the melt or superheated state of water by heating and cooling to 240 and 180 °C, respectively. Using a Linkam TMS 94 temperature controller, samples were heated at 10 °C/min and isothermal periods of 1 min were employed at the maximum experimental temperature to minimize hydrolytic degradation.^21^ For annealing experiments below the dissolution temperature, i.e., at 135, 140, and 145 °C, 3 min isothermal condition was applied. A standard rat-tail tendon collagen fiber was used to calibrate the modulus of the scattering vector at low *q.* A 300 K-W linear Pilatus detector (254 mm × 33.5 mm active area) was used to collect two-dimensional WAXD patterns with 12 s exposure time. SAXS images were collected with a 2D Pilatus 1 M detector (169 mm × 179 mm active area) placed at 3.0 m distance from the sample. All patterns were background-corrected, normalized for synchrotron beam fluctuations using an ionization chamber placed before the sample, and azimuthally integrated to give intensity against the scattering vector *q*. To emphasize not only variations in lattice planes but also in crystallinity, the signal of the purely amorphous phase (melt or dissolved state) was subtracted with a fixed factor. The relation *d = 2*π*/q* was used to convert the scattering angle into d-spacing, where *q* = 4π sin θ/λ with θ being half of the scattering angle.

### 2.3 Laboratory Wide-Angle X-ray Diffraction (Cu Kα)

2D wide-angle X-ray diffraction (WAXD) was carried out using a SAXSLAB Ganesha diffractometer with Cu Kα radiation (λ = 0.154 nm). The beam center and θ-range were calibrated via the diffraction pattern of silver behenate. The crystal perfection index (CPI) is based on the relative position of the observed (200) and (002) d-spacings against the d-spacings of the most thermodynamically stable monoclinic phase as observed in single crystals grown from solution.^35^ The CPI is thus calculated as follows where Ω = 0.194:
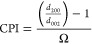


### 2.4 Solid-State ^1^H NMR Spectroscopy

The melt-quenched, defected monoclinic, and water-crystallized samples were followed on water uptake in their semicrystalline domains by *in situ* solid-state NMR spectroscopy. *In situ* variable-temperature ^1^H high-resolution magic angle spinning (HR-MAS) NMR experiments were performed on a Bruker DSX spectrometer operating at 500 MHz ^1^H Larmor frequency using a commercial MAS double-resonance probe for rotors with 4.0 mm outside diameter. The MAS spinning frequency was 10 kHz with a 4.0 μs π/2 pulse.

All samples were made by placing the different polymorphs in glass capillaries (Wilmad glass) with a filling of ∼10:90% in weight ratio and tightly sealed using a flame. During sample preparation, care was taken to avoid degradation or water evaporation. The temperature was controlled using a Bruker temperature control unit and ranged from 35 to 155 °C. The ^1^H HR-MAS NMR spectra were recorded every 10 °C. The ^1^H chemical shifts reported are relative to tetramethylsilane (TMS) using adamantane as an external reference.

### 2.5 Mechanical Testing

Prior to use, polyamide 6 granulate was dried overnight at 80 °C *in vacuo*. Cylindrical samples of 3 mm in diameter and 3 mm in height were machined from injection-molded tensile bars of 3 mm in thickness. The injection molding process, using a Boy XS with 100 kN clamping force, was characterized by melt and mold temperatures of 260 and 70 °C, respectively. To eliminate orientation effects, the injection-molded bars were above the glass transition temperature, 80 °C, *in vacuo* for 24 h. To preserve the polyamide 6 structure generated upon fast cooling, temperature elevation during machining was minimized by cold-water immersion. Tensile bars with ISO527 type 1A dimensions (width, 4 mm; height, 2 mm; length, 70 mm) were prepared by the above-mentioned injection molding conditions. Under the same temperature conditions, Izod impact bars with ISO 180 dimensions were prepared using an Xplore twin-screw microextruder of 5 mL in volume equipped with an Xplore Injection molder (5.5 mold).

In excess of water, samples for mechanical testing were exposed to superheated water in a temperature range of 105 to 145 °C for 30 min using 2–5 mL Biotage microwave reaction vials. Next, the samples were conditioned in a climate chamber at 25 °C with a relative humidity (RH) of 55% for 24 h. The effect of superheated water-induced structural changes on the mechanical properties was studied by compression, tensile, and Izod impact testing. All samples were dried at 80 °C *in vacuo* overnight prior to testing. Moreover, samples exposed to superheated water at 145 °C were additionally dried at 145 and 180 °C *in vacuo* for 30 min and slowly cooled to room temperature.

Compression tests were performed on the cylindrical samples using a Zwick Z100 100 kN universal tester at room temperature. Tests were performed on humidified samples and with a true strain rate of 10^–3^ s^–1^. True stresses were calculated in the assumption of incompressibility. Tensile testing was performed using a Zwick Z100 tensile tester equipped with a load cell of 10 kN. Before starting the measurements, a preload of 1 N was applied at a constant deformation rate of 5 mm/min at room temperature. The Izod impact test was performed on a Zwick HIT 5.5P. All mechanical tests performed were at least in fivefold.

### 2.6 Thermogravimetric Analysis

Weight loss due to evaporation of hydrated water and thermal degradation was studied using thermogravimetric analysis (TGA) using a TA Instruments Q500 apparatus. Approximately 15 g of sample was heated at 1 °C/min under a nitrogen atmosphere.

### 2.7 Differential Scanning Calorimetry

Differential scanning calorimetry (DSC) was performed on a TA Instruments Q200 DSC apparatus operating under a nitrogen atmosphere. Melting temperatures of polyamide 6 samples with different controlled crystallographic states were recorded by exposing a 3 mg ± 5% sample into aluminum pans to a heating ramp from 5 to 245 °C at 1 °C/min. For melting point depression by dissolution in the superheated state of water, the polyamide 6 samples were in TA large volume pans immersed in an equal weight of demineralized water, hermetically sealed and exposed to a ramp from 5 up to 175 °C at 1 °C/min.

## 3 Results and Discussion

### 3.1 Dissolution and Crystallization in the Presence of Superheated State of Water

**Figure 2:**
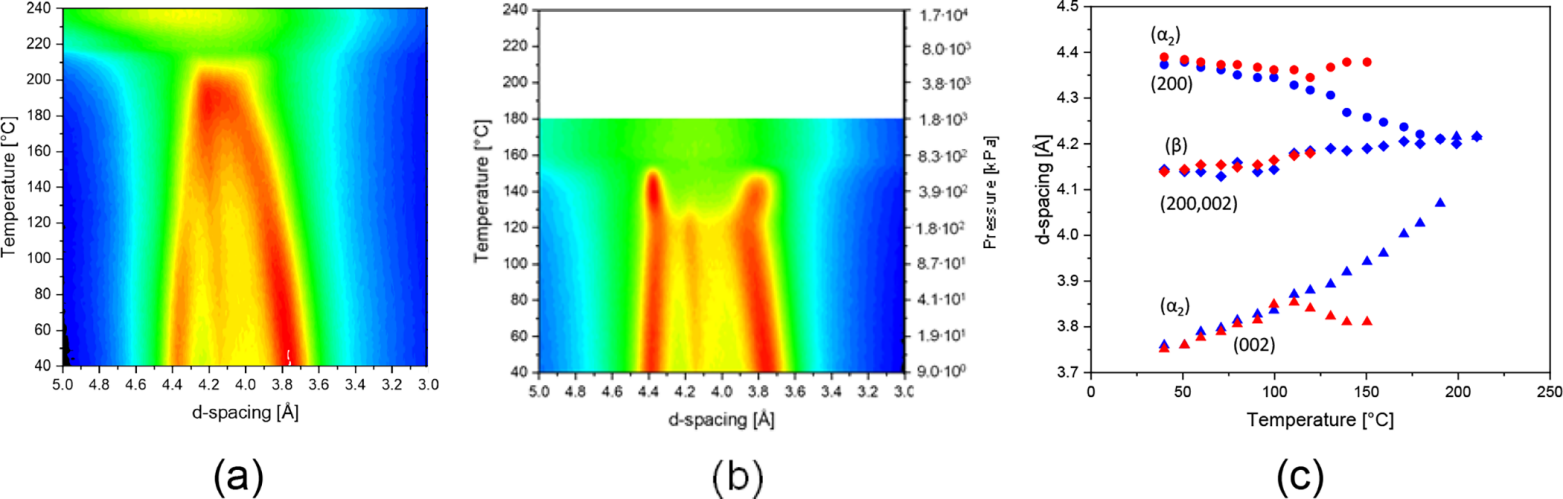
Intensity plots presenting the characteristic α_2_ and β d-spacings of melt-processed polyamide 6 as a function of temperature and corresponding water vapor pressure upon (a) melting and (b) structural refinement and successive dissolution in the superheated state of water. The temperature dependence of the characteristic lattice d-spacings, observed in panels (a) and (b), is summarized in panel (c). Blue and red symbols represent the lattice d-spacings upon heating without and with (superheated) water, respectively.

To understand the solubilization of melt-processed polyamide 6 in superheated water, the d-spacings are first monitored as a function of temperature in the absence of water. The trends are shown in Figure 2a, which are in agreement with earlier observations.^31^ To recall, at the start of the experiment, i.e., at 40 °C, the (200) and (002) diffraction signals of the monoclinic phase at 4.37 and 3.76 Å, respectively, and (200,002) of the pseudo-hexagonal β phase at 4.14 Å coexist. At 40 °C, the CPI of the melt-processed monoclinic phase is 0.836, reflecting an imperfect state, denoted by α_2_. The low CPI facilitates the appearance of the Brill transition at about 200 °C prior to melting (Figure 2c). Note that in polyamide 6, the Brill transition temperature of the monoclinic phase scales with the CPI. For the ultimately perfected monoclinic structure α_1_ (CPI = 1), the Brill transition temperature lies above the melting temperature.

**Figure 3:**
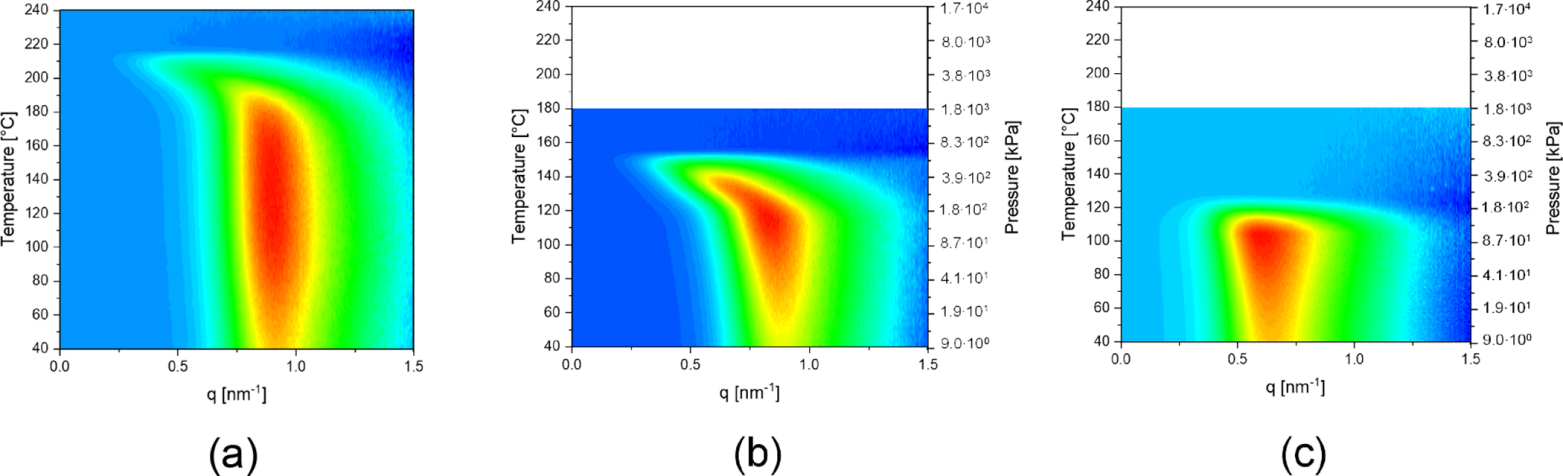
Small-angle X-ray scattering-derived *q*-values, representing the long period in the semicrystalline structure, of melt-crystallized PA6 (mc) upon (a) heating into the melt state, (b) heating and dissolution in water, and (c) crystallization while cooling from the superheated state of water.

Repetition of the experiment in the presence of water, as depicted in Figure 2b, reveals distinct crystallographic changes. The Bragg d-spacings as a function of temperature are summarized in Figure 2c. Independent of the presence of water, no crystallographic differences are observed up to 125 °C. However, in the presence of superheated water from 125 °C onward, the (200,002) β signal decreases in intensity and vanishes, while the intensity of both α_2_ diffraction signals increases. Prior to dissolution of the monoclinic crystals at 155 °C, confirmed by melting point depression in differential scanning calorimetry (Figure S2) and sharpening of the ^1^H HR-MAS signals of the polyamide in Figure 9, the d-spacings of the monoclinic intersheet and intrasheet diffraction signals separate progressively, resulting in the CPI approaching 1. The combination of these findings suggests a complete β → α_2_ transformation and successive α_2_ → α_1_ refinement. The SAXS patterns of the melt-processed polyamide 6 as a function of temperature in dry and water-immersed states are depicted in Figure 3a,b, respectively. In accordance with the WAXD signals in the sample without water (Figure 2a), no substantial changes prior to melting are observed in SAXS. Taking the crystallinity calculated from WAXD being 42% and a constant *q*-value of 7.7 nm results in a crystal thickness of 3.2 nm, which is representative of, on average, four repeat units that remain constant until the onset of melting at approximately 200 °C as observed by the decrease in peak intensity in WAXD. In the presence of water, with the substantial reorganization above 125 °C onward, the SAXS pattern (Figure 3b) shows a progressive increase in a long period. The increase in the long period, in combination with the enhanced WAXD intensity, suggests an increase in crystal thickness. The substantial increase in crystal thickness, prior to dissolution in water, supports structural modifications. Upon crystallization from the water–polymer solution, crystals having a lamellar thickness of 4.1 nm (six repeat units on average) are observed against the melt-crystallized ones with a lamellar thickness of 3.2 nm. Crystals grown from aqueous solution upon cooling (Figure 3c) possess similar lamellar thickness to the perfected crystals prior to the dissolution (Figure 3b). The CPI of the solution-grown crystals is found to be 1, similar to the crystals prior to the dissolution in the presence of water (Figure 2b).

Aiming for optimum mechanical performance, various postprocessing conditions to induce the β → α transformation have been studied *post mortem*, adopting timescales ranging from 1 to 24 h. The methodologies include annealing at elevated temperature solely, or in combination with hot water (100 °C), 20% formic acid solution, steam, or recrystallization from solution in superheated water at 150 °C.^40−44^*In situ* WAXD studies provide detailed insight into the dynamics of the transformation to optimally design the timescales of water-assisted postprocessing-shaped polyamide 6 technologies to render α, preferably the α_1_ phase, and thus ultimate performance. Based on similar *in situ* WAXD without the presence of water, Pepin et al. invalidate the proposed β → α transformation upon heating.^31^ At elevated temperatures, close to the melting temperature of polyamide 6, the gradual appearance of the intersheet (002) diffraction signal of the monoclinic phase is likely unresolved in the presence of the intense and typically broad (200,002) diffraction signal of the pseudo-hexagonal phase. The high diffusivity and shielding/plasticizing effect of superheated water molecules on polyamide crystals are expected to differ per crystallographic phase and may provide insight into the existence and origin of the β → α transition.

Based on our earlier correlation of the dissolution temperature of polyamides with the Brill transition,^13^ it can be suggested that above 100 °C, the β → α_2_ transformation in the presence of water is facilitated by the interaction of the mobile water molecules with the conformational changes and reduced hydrogen bonding efficiency present in the pseudo-hexagonal lattice. Figure 3b suggests that in specifically this temperature range, the pseudo-hexagonal phase of polyamide dissolves in the superheated state of water after which the polymer recrystallizes.

**Figure 4:**
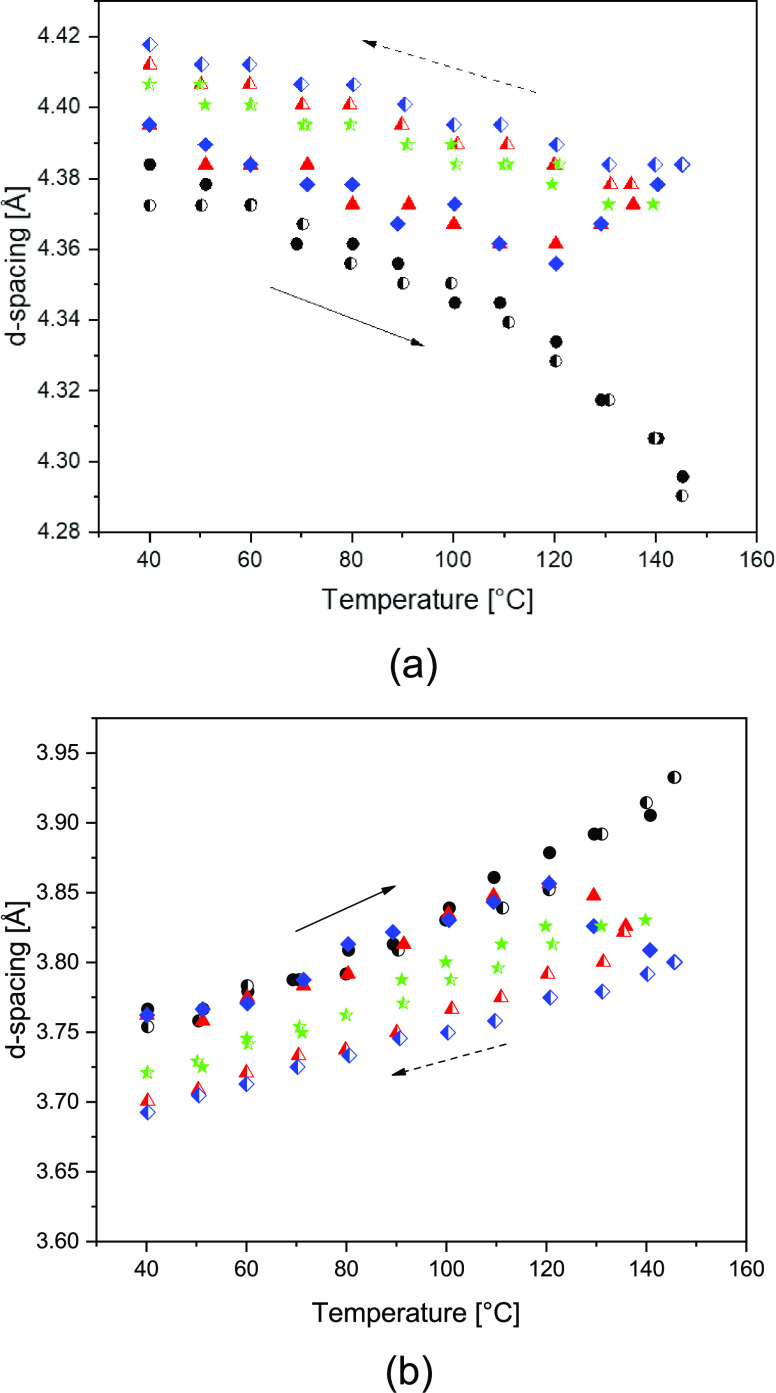
d-Spacings of (a) the intrasheet/interchain and (b) intersheet/interchain as a function of temperature upon exposure of melt-processed polyamide 6 without (black) and with water (red and blue) and water-crystallized perfected monoclinic polyamide 6 without water (green) to temperature cycles below the dissolution/Brill transition temperature. The closed and open symbols represent the heating and cooling ramps, respectively.

To unravel the role of water in the β to α_1_ (or α_2_ to α_1_) transformation, temperature cycles with and without water between 135 and 145 °C are performed. The annealing time at the chosen temperature was 3 min. In Figure 4, the d-spacings of the (a) intrasheet and (b) intersheet as a function of temperature during the temperature cycles are presented. Without water, both d-spacings of the melt-processed α_2_ phase follow the common reversible trend that precedes the Brill transition. To compare the water-mediated degree of crystal perfectioning, after the β → α_2_ transformation, the intra- and intersheet d-spacings of the water-crystallized α_1_ monoclinic phase in the same temperature range are shown in green in Figure 4. Only minor differences between the d-spacings upon heating and cooling are observed. Immersion of the melt-processed polyamide 6 in water induces a slight increase in intrasheet/interchain distance (Figure 4a). An increase in the intrasheet/interchain distance marks the improved uniplanar and spatial alignment of the amide motifs. Upon heating, both d-spacings follow the typical monoclinic trends, but from 120 °C, where the pseudo-hexagonal signal instantly disappears (Figure 2c), the d-values of the monoclinic signals start to diverge. The progressive nature, which continues up to melting, indicates continuous reorganization. The higher the annealing temperature, and the lower the degree of undercooling, the higher is the crystal perfection that is preserved upon cooling. In fact, the crystal perfection index of the monoclinic phase upon 3 min annealing at 135 and 145 °C increases from 0.865 to 0.988 and 1.00 in the wet state, respectively. To link the β → α transformation to the interaction of water molecules with amide moieties and increased conformational motion in the polymer close to the Brill transition, what follows are the *in situ* WAXD experiments performed on polyamide 6 initially composed of the pseudo-hexagonal phase.

### 3.2 Water Dissolution and the Brill Transition of the Pseudo-Hexagonal Lattice

**Figure 5:**
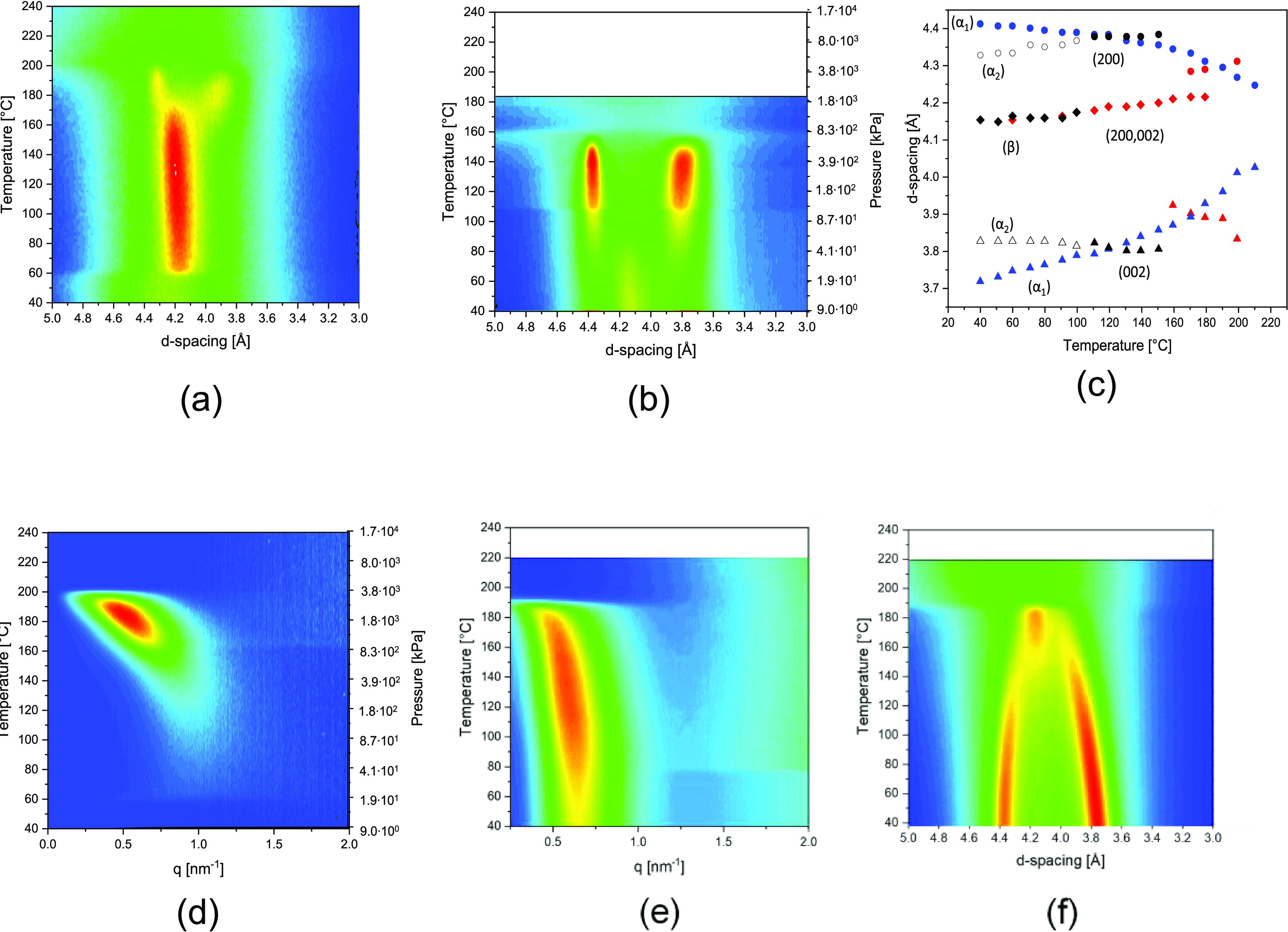
Structural and crystallographic changes of pseudo-hexagonal polyamide 6 without and with water. Wide-angle X-ray diffraction intensity plots upon heating a quenched polyamide 6 (a) without and (b) with water. Mobility either induced by heat (a) or water (b) induces crystallization into the pseudo-hexagonal phase. Trends of diffraction signals upon transformation of the pseudo-hexagonal phase (red) without water and the faintly existing defected monoclinic (open symbols) and pseudo-hexagonal (closed symbols) phases (black) in water are compared to the perfected monoclinic phase (blue) as a function of temperature (and corresponding water vapor pressure) in panel (c). Small-angle X-ray scattering patterns depicting the long period of the pseudo-hexagonal polyamide 6 that, (d) in water, reorganizes into the perfected monoclinic phase and, (e) after melt crystallization upon cooling, goes through the Brill transition as observed by WAXD (f).

Prior to the discussion on the role of superheated water in the structural changes and aqueous solubility of the pseudo-hexagonal phase upon heating, WAXD of a quenched polyamide 6 upon heating at 10 °C/min is recorded (Figure 5a). Below the glass transition temperature, i.e., below 50 °C, the melt-quenched polyamide 6 appears to be amorphous. Once the temperature approaches the glass transition temperature, polyamide 6 crystallizes into the pseudo-hexagonal phase with a characteristic single broad diffraction signal at 4.15 Å, which on heating at 150 °C, shifts to 4.21 Å. The broad diffraction signal is indicative of the disordered pseudo-hexagonal phase obtained at extreme undercooling.

From 150 °C onward, a shoulder at lower d-spacing appears, followed by the appearance of a second less deconvoluted diffraction signal at higher d-spacing and the complete disappearance of the pseudo-hexagonal diffraction signal at 170 °C. In Figure 5c, the d-spacing of the pseudo-hexagonal phase (200,002) as a function of temperature is plotted in red. As a comparative example, the (200) and (002) d-spacings of a dried water-crystallized α_1_ phase are shown in blue. Just above the crystal transformation, the d-spacings of the two diffraction signals adopt monoclinic values. Thus, the diffraction signals are assigned to the interchain/intrasheet (200) and interchain/intersheet (002) spacings, demonstrating a β → α transformation without water. The successive divergence of the two diffraction signals indicates progressive crystal perfectioning until 145 °C instead of the conventional convergence of the lattice d-spacings prior to melting.^31^

Based on annealing experiments of polyamide 6 (fibers) in combination with *post mortem* X-ray diffraction and spectroscopy studies, the α/β ratio is reported to increase.^2,34^ Time-resolved WAXD studies of Androsch et al., promoting a direct β → α transformation instead of a β → amorphous → α transformation, contradict the conclusion of Pepin et al. who reported a stable pseudo-hexagonal phase up to 190 °C that only adopts the monoclinic packing upon successive cooling. The stability of the pseudo-hexagonal phase up to 200 °C in the melt-processed sample (Figure 2a,c) and β → α transformation in the range of 160–180 °C in the cold crystallized sample (Figure 5a,c) indicates that the structural behavior of the pseudo-hexagonal phase as a function of temperature may not be generalized. The SAXS patterns of the amorphous polyamide 6 during heating, cold crystallization, β → α transformation, and final melting (Figure 5d) reveal the absence of long-range order within the respective *q*-range until the crystallographic transformation occurs. However, the SAXS patterns of the pseudo-hexagonal phase that precedes the Brill transition during slow crystallization from the melt (Figure 5e,f) do reveal a peak at *q* = 0.39 nm^–1^. It is likely that although the interchain spacing of differently generated pseudo-hexagonal phases is similar, their thermodynamic stability and potential transformation into the monoclinic phase depend on the size and an apparent perfection index of the crystal aggregates. Due to the broadness of the pseudo-hexagonal phase and the proximity of the transformation close to the melting temperature, causing the inter- and intrasheet spacings to be close to each other, deconvolution of the WAXD patterns to study the mechanism closely is challenged. The fact that the monoclinic d-spacings progressively diverge in Figure 2b suggests that the β → α transformation indeed occurs via intermediate amorphous states. In the following paragraph, we will also make use of water and its suppressing effect on thermally induced crystal-to-liquid transition temperature in polyamides and will provide further insight into the mechanism of the β → α transformation in polyamide 6 upon heating.

Upon immersion of the amorphous polyamide 6 in water, the interaction of water molecules with the amide motifs is immediately apparent (Figure 5b). Prior to heating, i.e., at 40 °C, WAXD reveals a more intense β diffraction signal at 4.15 Å and two weak shoulders at 4.33 and 3.83 Å that represent a monoclinic phase. Water is known to hydrate the amide moieties of the amorphous phase, lowering the glass transition temperature,^45^ providing sufficient molecular motion at 40 °C to facilitate cold crystallization, albeit the overall crystallinity is relatively low. Upon heating, from 70 °C onward, the intensity of the pseudo-hexagonal diffraction signal at 4.16 Å diminishes. The intensity of the diffraction peaks of the monoclinic phase (Figure 5b) increases above 90 °C, stressing the dissolution or amorphization of the pseudo-hexagonal β phase after which the monoclinic α phase is formed. The d-spacings of the intrasheet/interchain and intersheet/interchain diffraction signals instantly adopt values of the perfected monoclinic phase, which remain preserved until dissolution. Complete dissolution of polyamide 6 at about 155 °C was confirmed by melting point depression in DSC (Figure S2). The effect of the superheated water-induced structural refinement and its potential technological advances in terms of mechanical properties are addressed in the following section.

### 3.3 Crystal Perfection, Crystal Hydration, and Mechanical Properties

**Figure 6:**
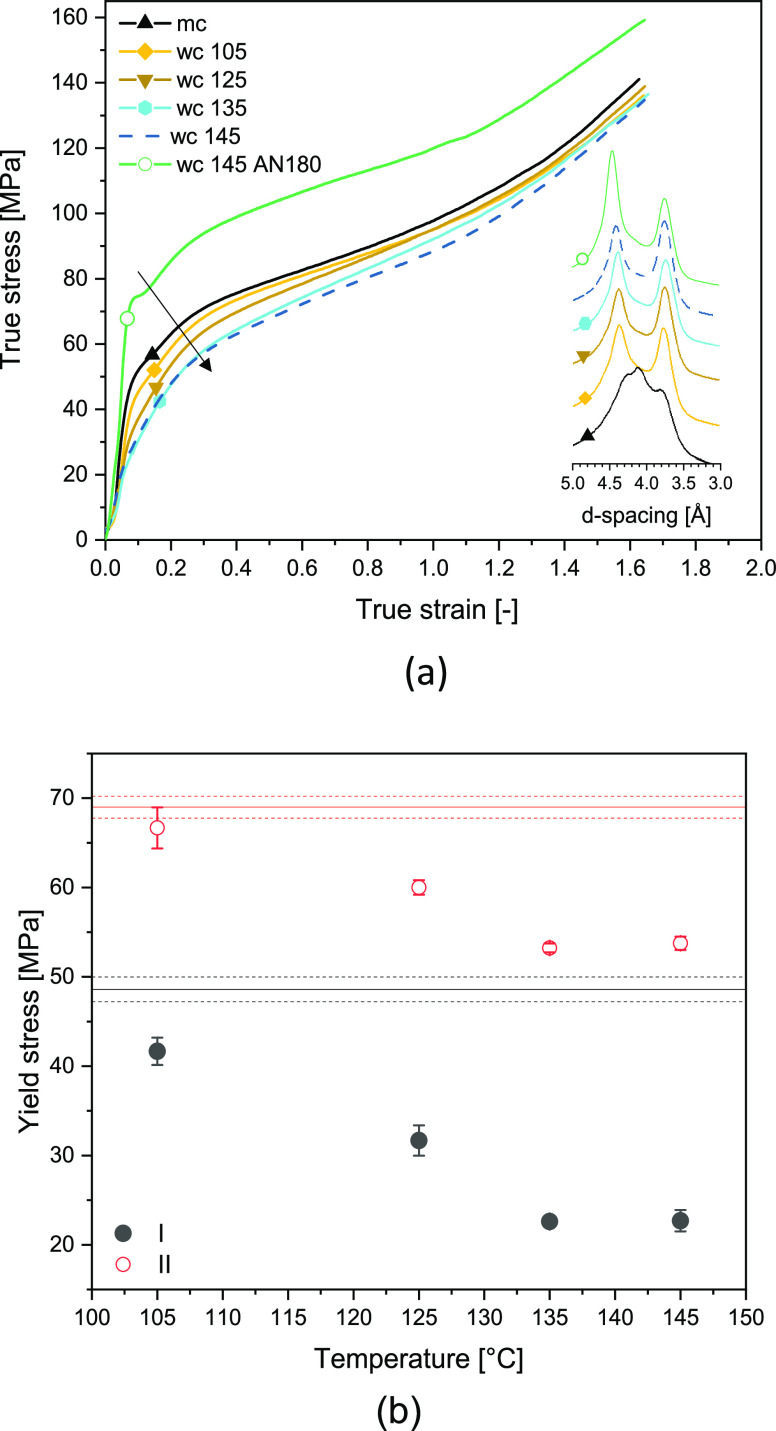
(a) Compression testing and wide-angle X-ray diffraction patterns of melt-crystallized polyamide 6 (mc) exposed to superheated water treatment (wc) of different temperatures ranging from 105 to 145 °C. Also, compression testing and X-ray diffraction patterns of polyamide 6 (β and α_2_) that is successively perfected in superheated water at 145 °C and dried at 80 °C (blue) and 180 °C (green) under vacuum. (b) First (I) and second (II) yield points after superheated water treatment at different temperatures.

Melt-crystallized (mc) polyamide 6 samples were annealed in the superheated state of water at temperatures ranging from 105 to 145 °C for 30 min. To eliminate the effect of variation in the hydration of the amorphous phase, all samples were conditioned at 55% relative humidity overnight to secure equal plasticization. Additionally, as hydrolytic degradation may entail plasticization effects by low molecular weight polyamide fractions, we have proven that no changes in the average molecular weights and polydispersity index occur within the experimental temperature range from 105 to 145 °C. Molecular weights and polydispersity indices are reported in Figure S6 and Table S2 of the Supporting Information. In Figure 6a, the stress–strain response of the crystallographically refined water-crystallized (wc) samples is presented. Despite the structural perfection (Figure 6a, inlay), the yield stress of the water-crystallized samples decreases. Yielding occurs in two stages as is evident from the first and second yield points. The first yield point at about 0.1 strain relates to stress-induced mobilization of the amorphous phase, whereas the second yield point in the strain range from 0.25 to 0.30 originates in the breakup of crystalline lamella.^45^ From the analysis of the yield points, given in Table S1 and Figure S3 of the Supporting Information, it appears that the strain for both yield points barely changes. The initial modulus (Table S1) and both yield stresses decrease despite the equilibration of the hydrated amorphous state, which in combination with the structural refinement, suggests that also the crystalline phase is plasticized upon treatment in the superheated state of water. Increasing the temperature of superheated water to above 135 °C does not induce a further decrease in yield stresses (Figure 6b). It is worth noting that on annealing the samples, in the presence of water, from 130 °C onward, the pseudo-hexagonal phase has fully transformed into the thermodynamically stable monoclinic phase (Figure 2c and inlay of Figure 6a). Plasticization of the crystalline phase suggests that water resides within the polyamide crystal lattice as for the first time detected by our group using solid-state ^1^H NMR spectroscopy.^21^

The effect of water on the crystallographic and mechanical aspects of the crystalline region is not well understood, specifically in relation to its exact location discriminating, for example, water on the crystal surface or within the crystalline lattice. Boukal reported that after exposing polyamide 6 to boiling water, hydration of the amorphous phase alone could not account for the observed macroscopic plasticization effect.^46^ The elastic moduli of the lattice in hydrated and nonhydrated forms remained identical. A slight reversible increase of intrasheet/interchain and a decrease in intersheet/interchain distance were observed upon hydration. The origin was assigned to water-induced swelling in the intracrystalline region but not the penetration of water in the lattice. In an earlier NMR study discriminating water in the interlamellar, interfibrillar, and void regions of polyamide 6 fibers based on different T1ρ relaxation times, Murthy attributed the lowering of the glass and Brill transition temperatures, structural transitions, and mechanical plasticization to hydration-enhanced chain mobility in the amorphous domains that effectively transfers to the crystalline domains via direct linkages.^47^ Miri et al. proposed an explanation for the mechanical plasticization, realizing that water that resides in the amorphous phase, or on the crystal surface, may penetrate the crystals upon deformation via stacking faults that are preferred loci for crystal slip and fragmentation.^32^

**Figure 7:**
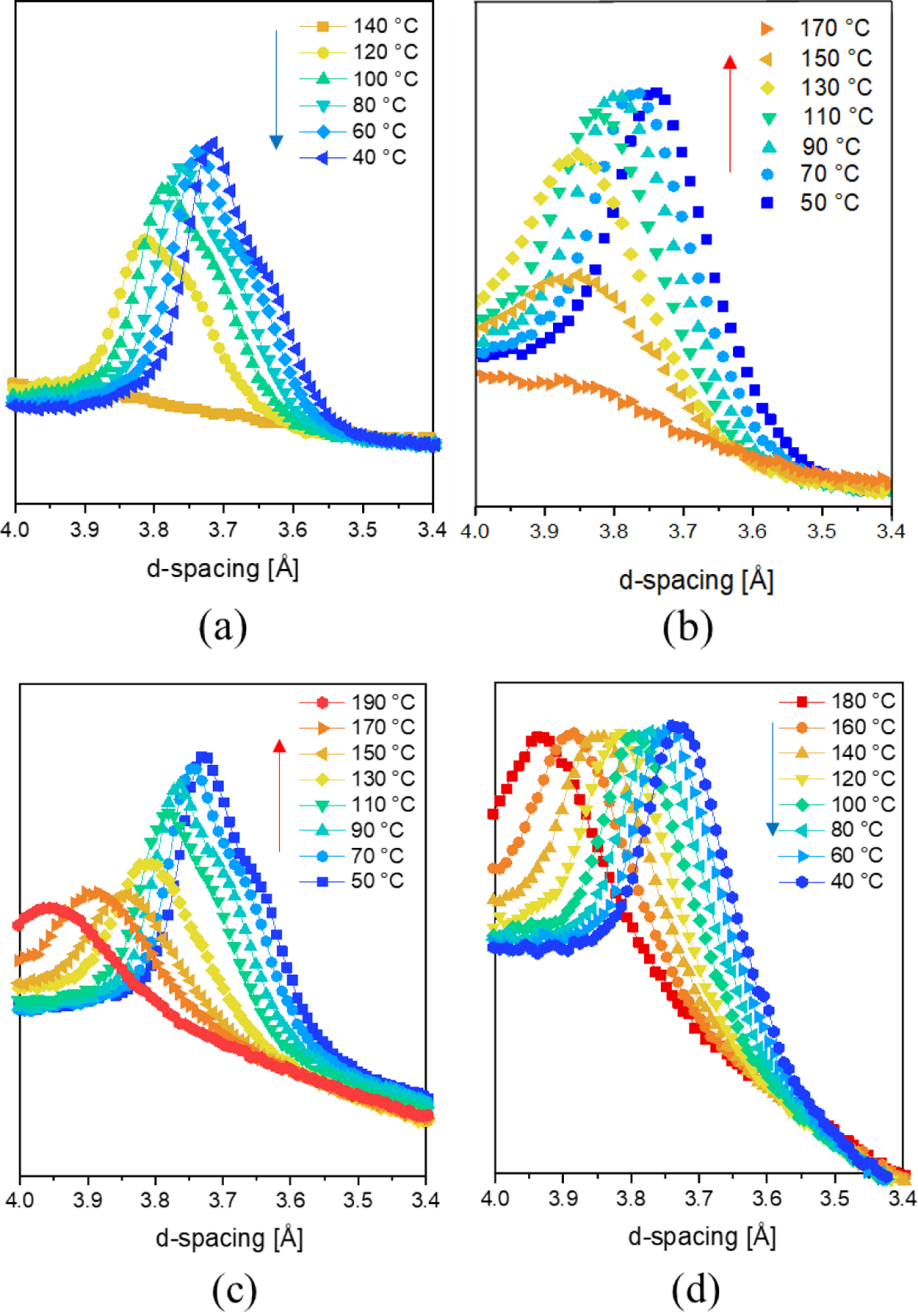
Intersheet (002)/(202) d-spacing recorded during (a) crystallization from the superheated state of water, (b) slow heating of the melt-crystallized monoclinic α_2_ sample (without water to the molten state), (c) heating of the water-crystallized α_1_ sample obtained from cycle (a) (without water close to the Brill transition without melting), and (d) cooling of the sample obtained from cycle (c) that was annealed close to the Brill transition temperature.

During crystallization from the superheated state of water, a shoulder appears in the intersheet/intrachain diffraction signal with lower d-spacing, 3.65 Å (Figure 7a). This shoulder appears at 120 °C and increases in intensity upon further crystallization. To elucidate whether the shoulder is attributed to hydration of the amorphous phase or crystal surface, a slowly cooled defected monoclinic sample is heated until dissolution in the superheated state of water (Figure 7b). Neither crystal transition nor the appearance of the shoulder of the (002)/(202) diffraction signal is observed in the superheated state. Only at the onset of dissolution, i.e., at 170 °C, the shoulder weakly appears. On reheating the superheated water-crystallized α_1_ sample up to 190 °C, the shoulder irreversibly disappears at ∼150 °C (Figure 7c,d). It is worth noting that although the perfected monoclinic α_1_ crystal does not show a Brill transition prior to melting, the convergence of the d-spacing before melting, which is followed up to 145 °C in Figure 4, arises due to a high amplitude of heterogeneous methylene conformers. The decreased intersheet/intrachain d-spacing in the α_1_ phase thus only arises upon the dissolution and crystallization in the presence of water. To recall, in an FTIR study using low molecular weight bisamide model crystals to exclude the complexity of the amorphous phase, the presence of water molecules in the lattice was found to shield the hydrogen bonding of the amide moieties. The shielding effect releases the conformational strain of the covalently linked amide moieties to the adjacent methylene units that exist in the energy-optimized solid state.^25,26^ This release allows energetically optimized chain conformations to promote the uniplanarity of the “hydrogen-bonded” sheets, expressed by a decrease in intersheet/intrachain distance. The fact that the shoulder coexists next to the conventional intrasheet/intrachain d-spacing (Figure 7a,c) suggests also that both water-shielded and nonshielded crystalline fractions exist. To investigate the crystal hydration for the different polyamide 6 crystallographic phases and its effect on crystallographic transformations and mechanical plasticization of the crystals, temperature-dependent ^1^H HR-MAS NMR spectroscopy in sealed capillaries is performed.

**Figure 8:**
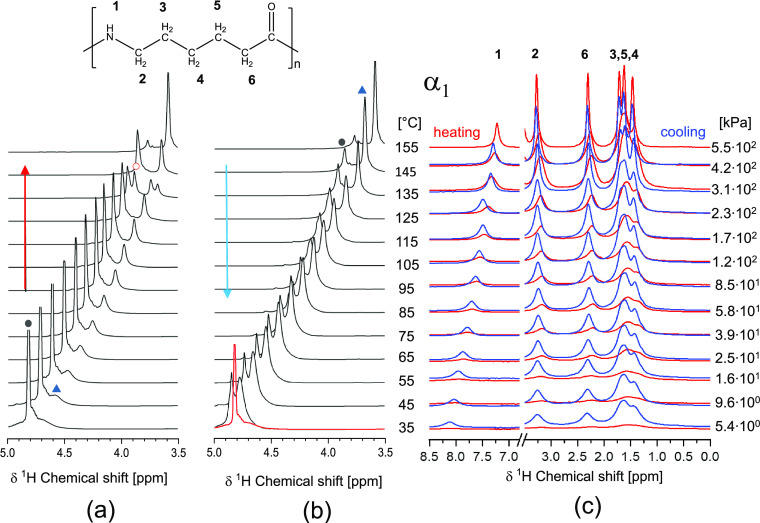
Temperature-dependent ^1^H HR-MAS spectra of the (a, b) water and (c) polyamide 6 protons upon heating and dissolution, and cooling and crystallization of perfected monoclinic α_1_ polyamide 6 in and from the superheated state of water, with the black solid circle assigned to bulk water, the red open circle assigned to water in the crystal lattice, and the blue up-pointing triangle assigned to water in the amorphous phases.

Upon heating the polyamide 6 thermodynamically favorable α_1_ phase, no structural modifications were observed even until dissolution in the superheated state of water (Figure 2a). The ^1^H NMR MAS spectrum of the perfected monoclinic α_1_ polyamide 6 immersed in water at room temperature (Figure 8a) reveals two ^1^H chemical shifts associated with the water molecules: an intense sharp peak at 4.85 ppm associated with the dynamically hydrogen-bonded water molecules in the bulk and a less intense relatively broad peak at 4.70 ppm. The lower chemical shift and broad line width indicate that these protons are of water molecules in a different chemical environment, tentatively localized in the amorphous phase of polyamide 6. Upon heating, both water ^1^H HR-MAS signals decrease in chemical shift. The shifts are paralleled by the sharpening of the aliphatic proton signals in the lower spectral regime (Figure 8c, red), which is indicative of reduced hydrogen bonding efficiency and increased conformational mobility.^13^ The peak intensity of water in the amorphous phase of polyamide 6 increases on the expense of the bulk water peak, stressing an increased hydration state of the amorphous domains. Close to dissolution, at 135 °C, two additional ^1^H signals appear next to the ones representing water in the bulk (3.93 ppm) and the amorphous phase of polyamide 6 (3.71 ppm). The peak at 3.87 ppm was previously assigned to water residing within the crystal lattice.^13,21^ Studying the chemical shifts as a function of temperature (Figure S4) shows that there is a new peak at 3.69 ppm that predominantly marks the solubilized state at 155 °C. It is also worth noting that prior to dissolution, with the migration of water into the lattice from 135 °C onward, the ^1^H HR-MAS signals of the methylene units adjacent to the amide motifs (2 and 6) sharpen and shift to higher ppm. These shifts indicate relaxation of the local conformational strain by shielding of the amide–amide hydrogen bonding in the crystals by water.^25^ Also, the ^1^H HR-MAS signals of the central methylene groups (3, 4, and 5) sharpen progressively and become more resolved, demonstrating high conformational mobility prior to dissolution. When crystallization in the superheated state occurs upon cooling (Figure 8b and Figure S3), three different chemical environments for water protons prevail at 35 °C, representing water in the bulk state and the amorphous and crystalline states of polyamide 6. In comparison to the perfected monoclinic α_1_ sample prior to the hydrothermal dissolution, at 35 °C, the retained sharp ^1^H HR-MAS signals of the polyamide and increased chemical shifts of the methylene units next to the amide motifs (unconstrained) upon structural refinement support the hydration of the amorphous and crystalline phases.

**Figure 9:**
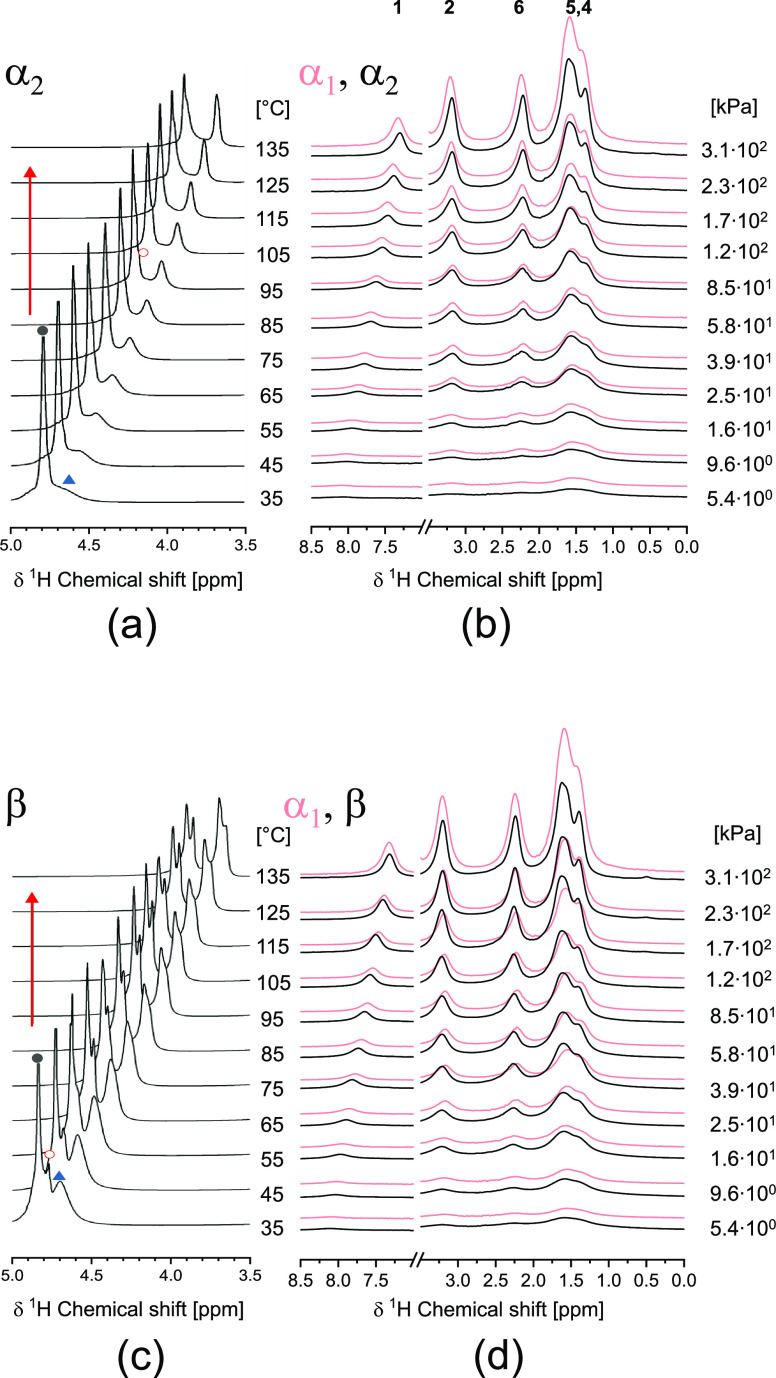
Temperature-dependent ^1^H HR-MAS spectra of water and polyamide 6 protons of (a, b) defected monoclinic α_2_ polyamide 6 and (c, d) initially amorphous polyamide 6 with, in red, the perfected monoclinic α_1_ as a reference upon heating into and annealing in the superheated state of water, with the black solid circle assigned to bulk water, the red open circle assigned to water in the crystal lattice, and the blue up-pointing triangle assigned to water in the amorphous phases.

The ^1^H HR-MAS NMR spectra of defected monoclinic α_2_ polyamide 6 in water, which are included in Figure 9a and Figure S3, show the peaks of water in its bulk state and the amorphous phase of polyamide 6. At 105 °C, the peak of water in the crystalline phase appears at 4.23 ppm, which increases in intensity upon further heating. The peak at the lower chemical shift adopts an antisymmetric shape, being indicative of a fourth chemical environment of water, likely originating from a small-solubilized fraction. The effect is more pronounced from 125 °C onward when, in WAXD, the crystallographic transformation of the pseudo-hexagonal β and defected monoclinic α_2_ phases into the perfected monoclinic α_1_ crystallographic form is observed. Besides the sharpening of the ^1^H HR-MAS signals of polyamide 6 due to the increased conformational mobility upon heating, no distinct spectral changes are observed (Figure 9b). Although, here, full dissolution of polyamide 6 in the superheated state of water does not occur (Figure 9a), water remains included in the lattice upon cooling (Figure S4), shielding the amide moieties, releasing the conformational strain, and decreasing the energetically optimized intersheet/intrachain d-spacing and mechanical plasticization. The ^1^H HR-MAS spectrum of the initially amorphous polyamide 6 in water depicted in Figure 9c reveals the presence of a third peak at 4.72 ppm at the start of the experiment. As this ^1^H HR-MAS signal is assigned to the water residing in the crystal lattice and causing the simultaneously increased chemical shift of the methylene protons next to the amide motifs (Figure 9d), inclusion of water in the crystal lattice is not only observed after crystallization in the superheated state of water. Hydration of polyamide 6 crystals is a result of crystallization in water even well below 100 °C. The asymmetric shape of the low ppm ^1^H HR-MAS signal becomes progressively more apparent from 75 °C onward, confirming that the crystal β → α_1_ transformation occurs via a solubilized state. It also implies that hydration of the crystalline amide moieties reduces the dissolution temperature of the pseudo-hexagonal polyamide 6 (crystallized in water) below 100 °C.

### 3.4 Restoration of Amide–Amide Hydrogen Bonding

Vinken et al. concluded for polyamide 46 that the water included in the crystal lattice can only be removed by heating the water-crystallized polyamide 46, which is a highly perfected monoclinic (α_1_) phase, above the Brill transition temperature of 205 °C.^21^ In the same study, it was also observed that by annealing the water crystallized above the Brill transition temperature, the perfected lattice d-spacings that match single-crystal values are irreversibly lost. A new question rises. Can structural refinement in the superheated state of water as post-treatment, or direct processing in the presence of water, technically be preserved for enhanced thermodynamic stability and mechanical performance despite crystal hydration? In perfected monoclinic α_1_ polyamide 6 crystals, the Brill transition temperature is above the melting temperature. Does the conformational motion in the crystallographic polyamide lattice that precedes the Brill transition provide sufficient motion to remove water from the lattice effectively? Melt-crystallized samples were annealed in the superheated state of water at 145 °C and subsequently dried at 80, 180, 190, and 200 °C *in vacuo*. To assure that no chemical degradation has occurred in the process, preservation of molecular weight was observed for all samples by GPC (Figures S6 and Table S3). The resulting thermogravimetric diagrams are given in Figure S5. For the sample dried at 80 °C, a gradual initial weight loss at 80 °C is followed by two successive declines in weight loss, starting at about 80 and 220 °C, respectively. The latter temperature matches the melting point and degradation onset of the perfected monoclinic polyamide 6. The first decline in weight loss, which is assigned to the removal of water from the amorphous phase of polyamide 6, disappears by drying close to the Brill transition temperature. With increased drying temperature, a smaller decrease in weight loss is observed.

**Figure 10:**
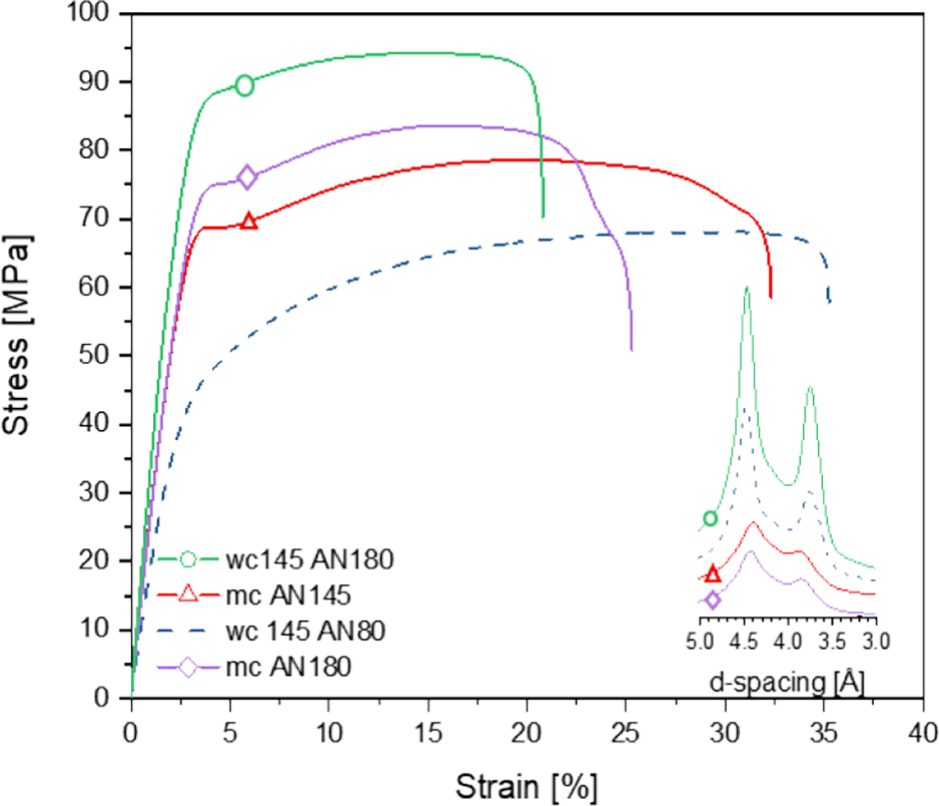
Tensile testing and wide-angle X-ray diffraction patterns of polyamide 6 annealed at 145 °C under vacuum or in the superheated state and successively dried at 180 °C under vacuum.

The true stress–strain diagram of the crystallographically perfected monoclinic α_1_ polyamide 6 treated at 145 °C and dried at 180 °C is included in Figure 6. Compression and WAXD (Figure 6, inlay) show that at 180 °C, water in the polyamide 6 crystal lattice can be removed, fostering the high crystal perfection of the monoclinic lattice and increasing the *E* modulus, yield stress(es), and toughness. Plasticization of the crystal lattice is also confirmed by the mechanical properties of injection-molded samples in tensile mode (Figure 10). The properties of polyamide 6 annealed at 145 °C under vacuum or in the superheated state and successively dried at 180 °C under vacuum are compared. The tensile data are complemented by the crystal perfection index (CPI) and Izod impact strength (Table 2). Despite the highest index of crystal perfection, crystal plasticization upon superheated water treatment at 145 °C and drying at 80 °C (wc 145AN80) leads to a decrease in *E* modulus and maximum stress of 19 and 22%, respectively, in comparison to the melt-crystallized sample annealed at 145 °C without water. The impact strength of the water-treated sample increases significantly to an extent that it does not break while using the employed pendula weight. Restoration of the amide–amide hydrogen bonding by the removal of water from the crystal lattice at 180 °C results in an *E* modulus of 3.37 GPa and a maximum stress of 87.1 MPa, which compared to the melt-crystallized sample, show substantial increases of 26 and 5.1%, respectively. Also, the impact strength increases with a notable difference of 168%.

**Table 2:** Crystal Perfection Index (CPI), Tensile Modulus (*E*), Maximum Tensile Stress (σ_max_), and Izod Impact Strength

sample	CPI	*E* (GPa)	σ_max_ (MPa)	impact strength (kJ/m^2^)
mc AN145	0.734	2.60 ± 0.2	77.7 ± 1.3	9.0 ± 2.3
mc AN180	0.779	2.67 ± 0.19	82.8 ± 1.9	10.0 ± 1.6
wc 145AN80	0.996	2.10 ± 0.18	60.9 ± 3.5	n.a.
wc 145AN180	0.996	3.37 ± 0.14	87.1 ± 4.5	26.8 ± 7.1

## 4 Conclusions

Monoclinic crystals in the most thermodynamically stable conformation show ultimate polyamide 6 mechanical and thermal performance. However, the high cooling rates intrinsic to melt shaping limit polyamide chains to timely adopt the all-trans chain conformations and ideally aligned hydrogen-bonding geometries, inhibiting the desired thermodynamically stable monoclinic packing. Without high shear or strain rates, melt shaping typically renders disordered gauche conformers and energetically misaligned hydrogen bonding, which are signature to the thermodynamically least stable crystallographic pseudo-hexagonal phase. At best, the pseudo-hexagonal phase coexists with a defected all-trans monoclinic phase featuring a small difference in intra- and intersheet d-spacings and yet energetically non-optimized hydrogen bonding alignment.

Upon heating polyamide 6 consisting of the coexisting defected monoclinic and pseudo-hexagonal phases into the superheated state of water, the pseudo-hexagonal phase dissolves and instantly recrystallizes into a defected monoclinic phase. The temperature window coincides with the Brill transition of the pseudo-hexagonal phase, which is defined by conformational changes instead of crystallographic changes. After the transition, further heating induces the perfectioning of the monoclinic phase and crystal thickening until dissolution occurs at 155 °C.

Like those of the monoclinic phase, the physical properties of the pseudo-hexagonal phase cannot be generalized. Dependent on its defected state, which originates from the degree of conformational disorder and misalignment of hydrogen bonding moieties, i.e., the crystallization conditions, structural transition temperatures vary. Heating or immersion in water of an initially amorphous polyamide 6 leads to cold crystallization and a highly defected pseudo-hexagonal state that, even without water, exhibits a pseudo-hexagonal to monoclinic transition via an intermediate liquid state and successive perfections. In the presence of water, instant cold crystallization at room temperature occurs, hydrating the crystal hydrogen bonding that lowers the dissolution temperature of the defected pseudo-hexagonal phase and recrystallization into perfected monoclinic phase to temperatures below 100 °C.

Without dissolution of the monoclinic phase, the water-induced refined structure prevails at room temperature, but hydration of crystalline hydrogen bonding plasticizes the crystals, decreasing Young’s modulus, yield, and breaking stress and increasing the impact strength. The inclusion of water molecules in the crystal lattice occurs during crystallization in the presence of water. Even during cold crystallization at room temperature, the presence of water molecules in the crystalline lattice is observed.

The data conclusively show that the aqueous solubility of polyamide 6 is correlated with the conformational disorder and hydrogen bonding efficiency in the lattice, intrinsically influencing the crystallographic forms and the Brill transition temperature. Using the water plasticization effect to achieve the thermodynamically stable polyamide 6 polymorph in shaped products, followed by its dehydration while maintaining the high crystal perfection, is the route to obtain the ultimate mechanical and thermal properties in polyamide 6. These findings are generic, having potential in optimization of the thermomechanical properties of not only all polyamides but also hydrogen-bonded synthetic and biobased polymeric materials overall.

## Supporting Information Available

Figure S1: melting point depression in the dissolution of various aliphatic polyamides in the superheated state of water; Figure S2: differential scanning calorimetry of polyamide 6 samples either being amorphous or controlled polymorphic in nature at room temperature as proven by wide-angle X-ray diffraction without and with water; Figure S3: examples of determination of yield stresses I and II by plotting the tangent on the true stress–true strain diagrams from compression testing; Table S1: Young’s modulus, yield stress I, and yield stress II derived from compression tests reporting true stress–true strain diagrams (Figure 5 and Figure S1) on melt-crystallized polyamide 6 (mc) treated in superheated water at 105, 125, 135, and 145 °C; Figure S4: temperature-dependent ^1^H HR-MAS spectra of water protons upon heating and dissolution and cooling and crystallization of perfected monoclinic α_1_ and defected monoclinic α_2_ phases and amorphous PA6 in and from the superheated state of water; Figure S5: thermogravimetric response of polyamide 6 treated in superheated water at 145 °C (wc145), annealed at different temperatures close to the Brill transition at 180, 190, and 200 °C; Figure S6: molar mass distribution of PA6 treated in superheated water at different temperatures; Table S2: number-average molecular weight (*M*_n_), weight-average molecular weight, and polydispersity as measured by GPC of melt-crystallized (mc) or superheated water-treated (wc) polyamide 6 at different temperatures ranging from 105 to 200 °C; Figure S7: molar mass distribution of PA6 water-crystallized in the superheated state (wc145) and annealed at 80 °C (wc 145 AN80) and 180 °C (wc145AN180) and melt-crystallized PA6 annealed at 80, 145, and 180 °C; Table S3: number-average molecular weight (*M*_n_), weight-average molecular weight (*M*_w_), and polydispersity as measured by GPC of melt-crystallized (mc) and superheated water-treated (wc) polyamide 6 dried/annealed (AN) at the given temperatures (PDF)
