# Supplementary material for: Hydration, Refinement, and Dissolution of the Crystalline Phase in Polyamide 6 Polymorphs for Ultimate Thermomechanical Properties
Source: Macromolecules. 2022 Jun 13;55(12):5080–93. doi: 10.1021/acs.macromol.2c00211 (PMC9245196; doi:10.1021/acs.macromol.2c00211)
Supplement: Supplementary file 1 — ma2c00211_si_001.pdf [file ma2c00211_si_001.pdf]

SUPPORTING INFORMATION

Hydration, refinement and dissolution of the crystalline phase in polyamide 6 polymorphs for ultimate thermo-mechanical properties

Milo Gardeniers[1], Mohanraj Mani[1], Ele de Boer[1], Daniel Hermida-Merino[2], Robert Graf[3], Sanjay Rastogi[1,4], Jules A.W. Harings[1]**

[1]Aachen-Maastricht Institute for Biobased Materials, Maastricht University, P.O. Box 616, 6200 MD, Maastricht, The Netherlands. [2]European Synchrotron Radiation Facility (ESRF), DUBBLE-CRG, FR-38043 Grenoble Cedex, France. [3]Max Planck Institute for Polymer Research, Ackermannweg 10, 55128 Mainz, Germany. [4] King Abdullah University of Science and Technology, 4700 KAUST, Thuwal, 23955-6900, Saudi Arabia.

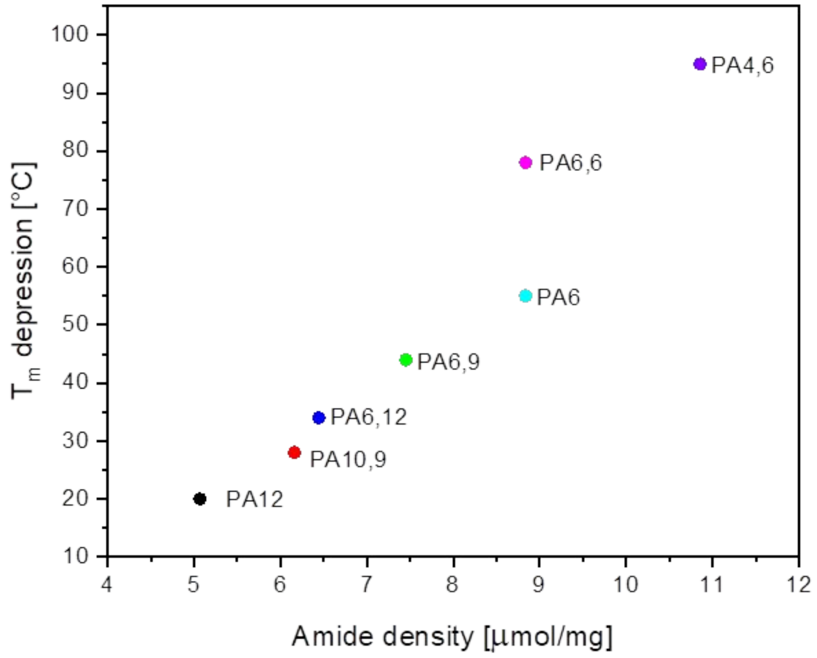

Figure S1: Melting point depression in the dissolution of various aliphatic polyamides in the superheated state of water. The data, taken from [5,21,29,30], was derived via hydrothermal DSC experiments taking polyamide weight fractions in the range of 20-70 wt-% where the dissolution temperature has been reported independent of polyamide weight fraction.

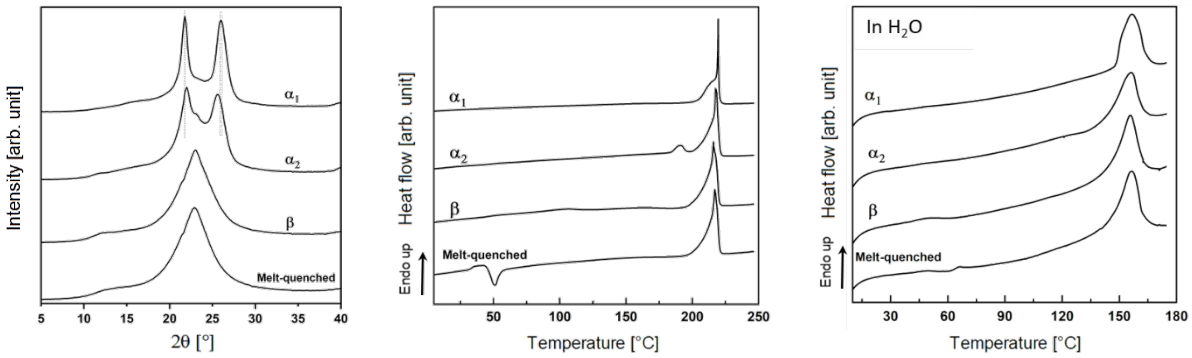

(a) (b) (c)

Figure S2: Differential scanning calorimetry of polyamide 6 samples either being amorphous or controlled polymorphic in nature at room temperature as proven by (a) Wide Angle X-ray Diffraction without (b) and with (c) water. The melting point depression in the presence of water follows the Flory-Huggins theory on the dissolution of a polymer in a good solvent and supports full dissolution of the semi-crystalline polyamide 6 in the superheated state of water.

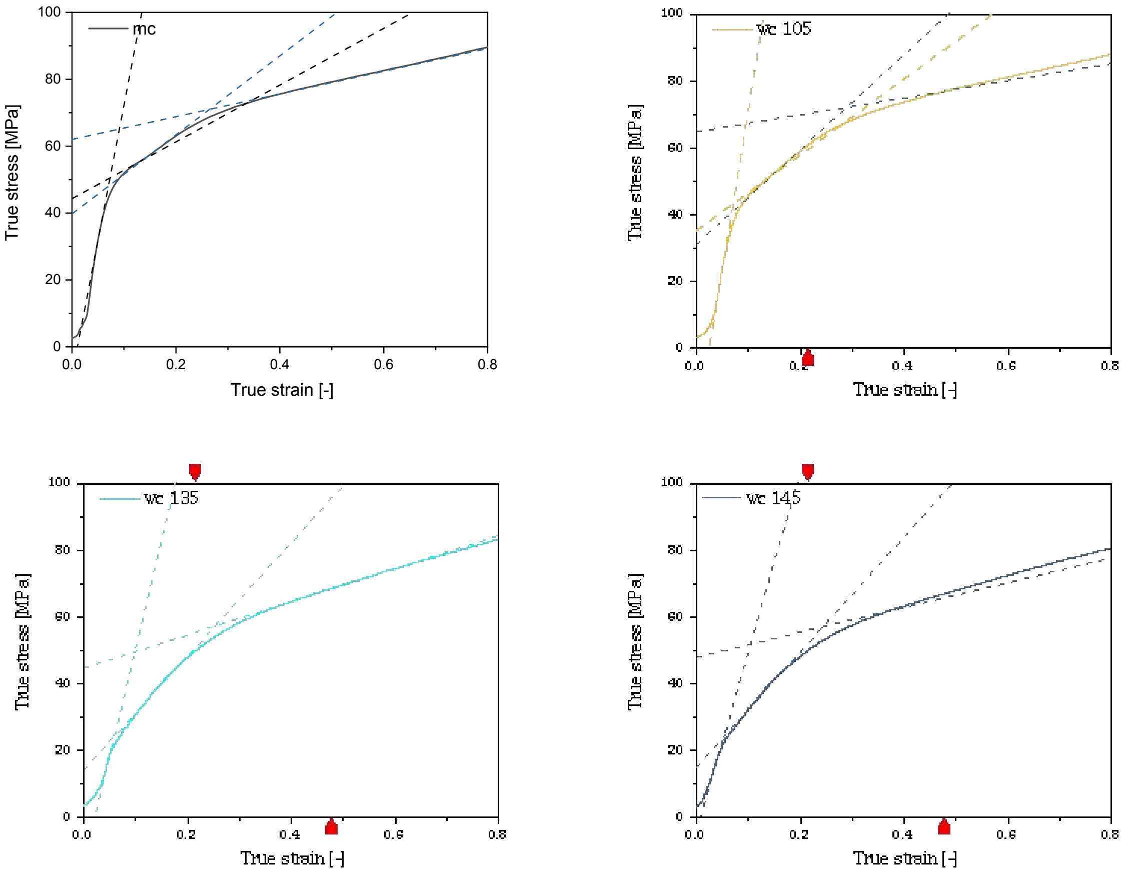

Figure S3: Examples of determination yield stress I and II by plotting tangent on the the true stress – true strain diagrams from compression testing. Sample coding represents polyamide 6 samples melt crystallized (mc) and the temperature of superheated water treatment (wc).

Table S1: Young's modulus, yield stress I and yield stress II derived from compression tests reporting true stress –true strain diagrams, Figure 5 and Figure S1, on melt crystallized polyamide 6 (mc) treated in superheated water at 105, 125, 135, and 145 °C (see sample coding). The sample treated in superheated water at 145 °C was additionally dried/annealed (AN) at 180 °C.

	**Modulus True**	**st. dev**	**Yield stress I**	**st. dev**	**Yield stress II**	**st. dev**
	GPa		MPa		MPa	
**mc**	1.04	± 0.12	49.5	± 1.0	69.5	±0.9
**wc 105**	0.99	± 0.02	41.7	± 1.5	66.8	± 2.3
**wc 125**	0.97	± 0.12	31.7	± 1.7	60.0	± 0.2
**wc135**	0.76	± 0.08	22.6	± 0.6	53.2	± 0.5
**wc145**	0.64	± 0.10	22.7	± 1.2	53.8	± 0.8
**wc145 AN180**	1.72	± 0.02	74.5	± 1.5	95.0	± 2.0

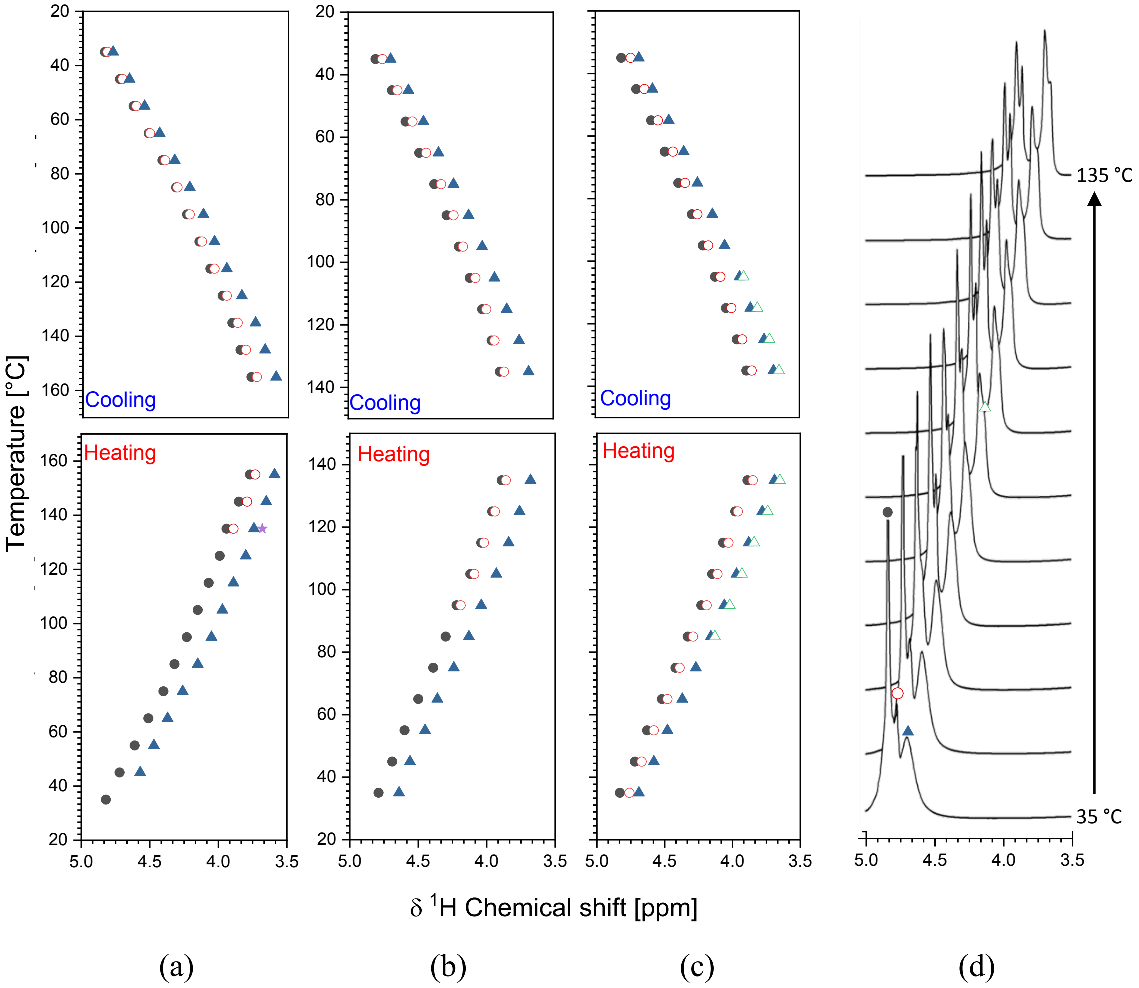

Figure S4: Temperature dependent ¹H HR MAS spectra following water protons upon heating and dissolution, and cooling and crystallization of (a) perfected monoclinic α_1, (b) defected monoclinic $\alpha_{2,}$ (c) amorphous PA6 in and from the superheated state of water. With ● assigned to bulk water, ○ assigned to water in crystal lattice, ▲ assigned to water in the amorphous phases, △ assigned to a shoulder existing upon heating close to the dissolution temperature of the pseudo-hexaganol phase. (d) Heating of amorphous PA6 from the superheated state.

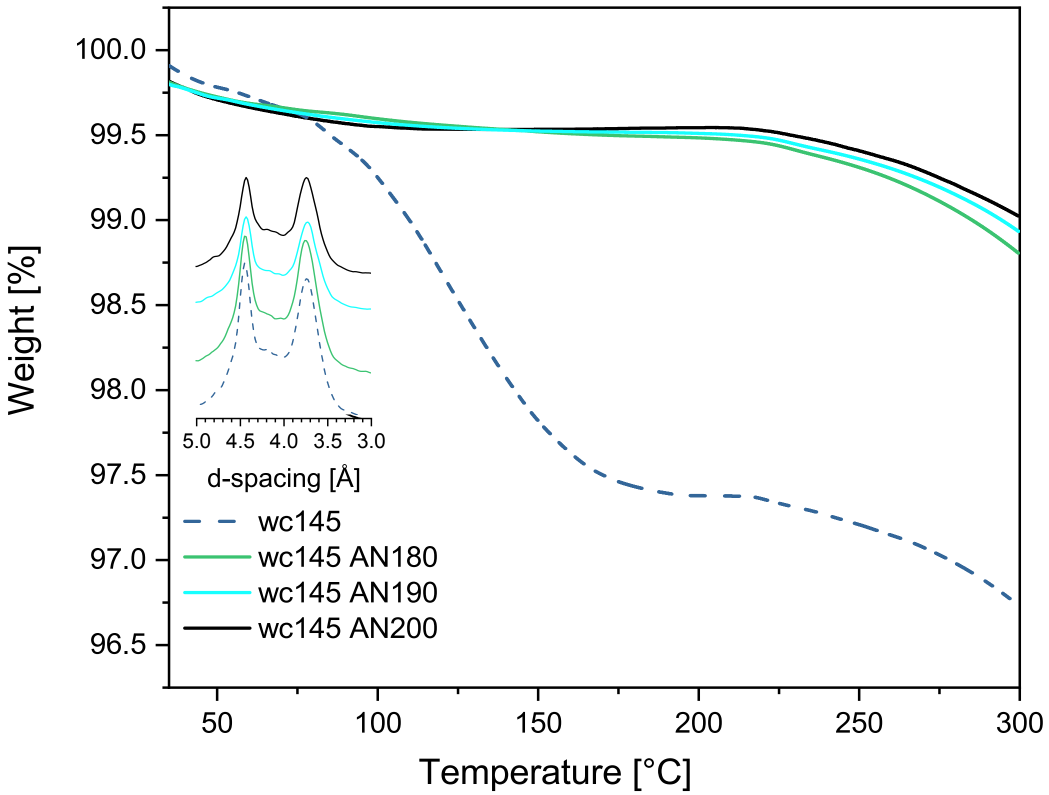

Figure S5: Thermogravimetric response of polyamide 6 treated in superheated water at 145 °C (wc145) annealed at different temperatures close to the Brill transition at 180, 190, 200 °C. Inlay shows monoclinic crystal structure of the treated samples by WAXD.

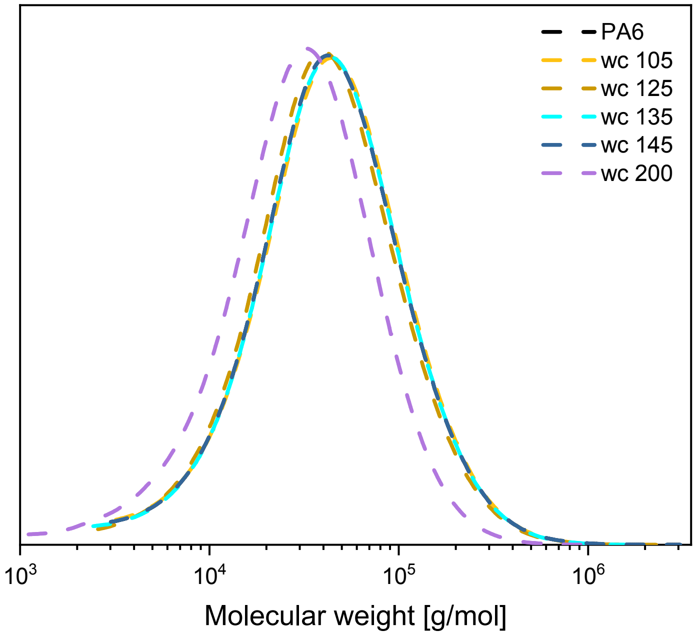

Figure S6: Molar mass distribution of PA6 treated in superheated water at different temperatures.

Table S2: Number average molecular weight (Mn), weight average molecular weight and polydispersity as measured by GPC of melt crystallized (mc) or superheated water treated (wc) polyamide 6 at different temperatures ranging from 105 to 200 °C.

Code	Mn [g/mol]	Mw [g/mol]	PDI
**PA6 mc**	28250	64350	2.3
**wc 105**	28700	64930	2.3
**wc 125**	26790	60670	2.3
**wc 135**	27480	63940	2.3
**wc 145**	28250	64350	2.3
**wc 200**	18160	43160	2.4

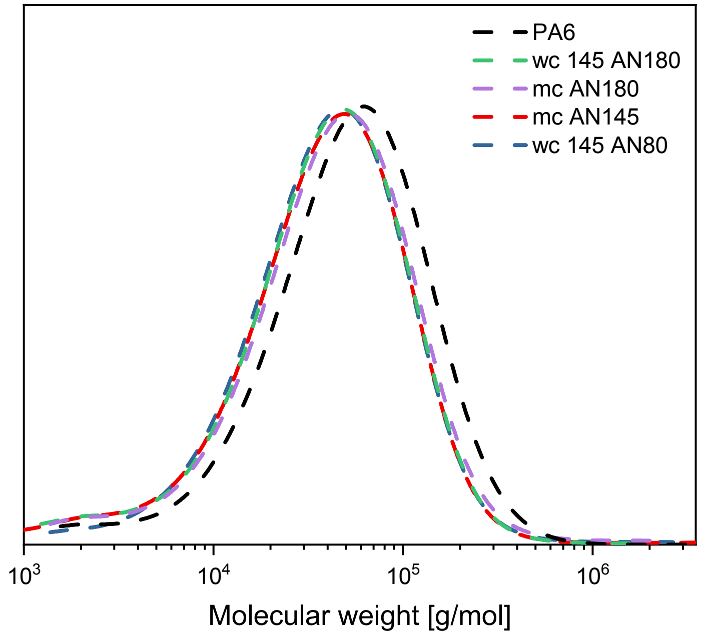

Figure S7: Molar mass distribution of PA6 water crystallized in the superheated state (wc145), annealed at 80 °C (wc 145 AN80), 180 °C (wc145AN180), and melt crystallized PA6 annealed at the following temperatures 80, 145, and 180 °C.

Table S3: Number average molecular weight (Mn), weight average molecular weight (Mw) and polydispersity as measured by GPC of melt crystallized (mc) and superheated water treated (wc) polyamide 6 and dried/annealed (AN) at the given temperatures (sample codes).

Code	Mn [g/mol]	Mw [g/mol]	PDI
PA6 mc	27740	77550	2.8
wc 145 AN180	20290	60470	2.9
mc AN180	21970	69930	3.2
mc AN145	18920	66210	3.5
wc 145 AN80	22970	64240	2.8
